# Foliar Elemental Analysis of Brazilian Crops via Portable X-ray Fluorescence Spectrometry

**DOI:** 10.3390/s20092509

**Published:** 2020-04-29

**Authors:** Camila S. Borges, David C. Weindorf, Geila S. Carvalho, Luiz R. G. Guilherme, Thalita Takayama, Nilton Curi, Geraldo J. E. O. Lima, Bruno T. Ribeiro

**Affiliations:** 1 Department of Soil Science, Federal University of Lavras – UFLA, Doutor Sylvio Menicucci Avenue, Lavras 37200-900, Minas Gerais State, Brazil; camila.borges@estudante.ufla.br (C.S.B.); geilacarvalho@ufla.br (G.S.C.); guilherm@ufla.br (L.R.G.G.); thalita.takayama@estudante.ufla.br (T.T.); niltcuri@ufla.br (N.C.); 2 Department of Plant and Soil Science, Texas Tech University, Bayer Plant Science Building, Room 211A, 2911 15th Street, Lubbock, TX 79409-2122, USA; david.weindorf@ttu.edu; 3 Campo – Environmental and Technological Agricultural Center, Lindolfo Garcia Adjuto Street, 1000, Paracatu 38600-000, Minas Gerais State, Brazil; geraldo.lima@campoanalises.com.br

**Keywords:** foliar analysis, plant nutrition, proximal sensors

## Abstract

Foliar analysis is very important for the nutritional management of crops and as a supplemental parameter for soil fertilizer recommendation. The elemental composition of plants is traditionally obtained by laboratory-based methods after acid digestion of ground and sieved leaf samples. This analysis is time-consuming and generates toxic waste. By comparison, portable X-ray fluorescence (pXRF) spectrometry is a promising technology for rapid characterization of plants, eliminating such constraints. This worked aimed to assess the pXRF performance for elemental quantification of leaf samples from important Brazilian crops. For that, 614 samples from 28 plant species were collected across different regions of Brazil. Ground and sieved samples were analyzed after acid digestion (AD), followed by quantification via inductively coupled plasma optical emission spectroscopy (ICP-OES) to determine the concentration of macronutrients (P, K, Ca, Mg, and S) and micronutrients (Fe, Zn, Mn, and Cu). The same plant nutrients were directly analyzed on ground leaf samples via pXRF. Four certified reference materials (CRMs) for plants were used for quality assurance control. Except for Mg, a very strong correlation was observed between pXRF and AD for all plant-nutrients and crops. The relationship between methods was nutrient- and crop-dependent. In particular, eucalyptus displayed optimal correlations for all elements, except for Mg. Opposite to eucalyptus, sugarcane showed the worst correlations for all the evaluated elements, except for S, which had a very strong correlation coefficient. Results demonstrate that for many crops, pXRF can reasonably quantify the concentration of macro- and micronutrients on ground and sieved leaf samples. Undoubtedly, this will contribute to enhance crop management strategies concomitant with increasing food quality and food security.

## 1. Introduction

The nutritional status of crops is crucial for assuring high productivity, food quality, and food security [1,2]. Well-nourished plants are more resilient to pests and diseases [3,4,5] and to adverse environmental conditions (e.g., dry season, soil water deficiency) [6,7]. At present, Brazil is widely recognized as a global food supplier [8] and for being one of the last agricultural frontiers. According to current world rankings [8,9], Brazil is the number 1 producer of soybean, sugarcane, and coffee; the number 2 producer of oilseeds (sunflower seed, peanuts (in shell), cottonseed, and rapeseed); and the number 3 producer of maize. Suitable management of soil fertility and plant mineral nutrition in Brazilian agriculture is one of the main factors responsible for increasing productivity and achieving food security goals in highly weathered-leached soils of Brazil [10].

The technical recommendation of fertilizers is frequently comprised of results from soil fertility analysis [11,12]. Additionally, chemical foliar analysis can be used as a complementary parameter for recommendation of fertilizers [13,14] to confirm observed visual deficiency of a given plant nutrient in the field and for temporal monitoring of the nutritional status of crops [15,16]. In sum, soil and foliar analyses are fundamental for successful fertilization and management of crops [17,18,19].

Foliar analysis of crops has been traditionally performed by wet digestion of oven-dried and ground leaf samples under laboratory conditions [20,21]. This analysis is time and labor consumptive and requires numerous chemicals. In the last two decades, portable X-ray fluorescence (pXRF) spectrometry has been an important and innovative tool in soil science [22,23]. At present, the pXRF method is recognized as an official method for soil analysis [22,23,24,25,26]. In a few seconds, under laboratory conditions or directly in the field, the total elemental composition of soils can be easily and adequately obtained. Furthermore, pXRF methods require no chemicals and are thus environmentally friendly. Based on pXRF spectra, prediction models of many soil properties (e.g., pH, texture, soil organic matter, macro- and micronutrients) have been established [27,28,29,30,31]. Several pXRF soil studies have been successfully conducted in tropical environments [32,33].

pXRF approaches can also be used for chemical analysis of plant tissues and seeds [34,35,36]. However, there is no standard method for this and more investigations are still needed. Some previous works have successfully employed the pXRF to assess the elemental composition of vegetation [37,38,39,40,41,42,43]. The concentrations of Cu, Zn, Pb, K, and Fe from different plant species growing in polluted mines were obtained via pXRF and were well correlated to laboratory-based methods [44]. For grasses, a strong correlation between pXRF and acid digestion was obtained for P, K, Ca, and Fe [45]. Sulfur, K, and Ca were reliably measured in cowpea, croton, mango, and maize leaves [40]. However, in Brazil, very few studies using pXRF for foliar analysis have been performed. The performance of pXRF was tested for soybean leaves [35] and for some varieties of sugarcane [46].

Given the lack of extensive research on pXRF elemental characterization of Brazilian crops, a study of such seems timely. Thus, the objective of this research was to compare the concentrations of macro- (P, K, Ca, Mg, and S) and micronutrients (Cu, Fe, Zn, and Mn) obtained via pXRF with those obtained via nitro-perchloric digestion, followed by inductively coupled plasma emission spectroscopy (ICP-OES) quantification. It was hypothesized that the pXRF will accurately quantify plant nutrients, showing strong correlations with conventional acid digestion and lab-based methods. These correlations are expected to be crop-dependent. If suitable correlations are obtained, the pXRF can be proposed as a fast, accurate, and environmentally friendly method for foliar analysis.

## 2. Material and Methods

### 2.1. Plant Material Collection from Brazilian Crops

**Figure 1:**
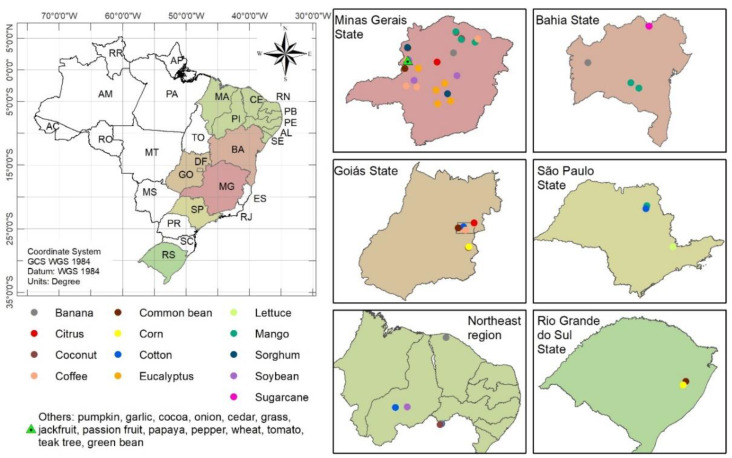
Location of crops in different regions of Brazil selected for this study.

Diagnostic leaves from 28 plant species were collected in different regions of Brazil (Southern, Southeastern, and Northeast) (Figure 1). The procedures for sampling the diagnostic leaves in the field were specific for each crop (Table S1) [11]. A total of 614 composite leaf samples were collected for this work. Table 1 shows the number of samples for each crop. The samples were sent to an ISO/IEC Standard 17025 Laboratory of Soil and Plant Analysis located in Minas Gerais State, Brazil. In the laboratory, the samples were carefully washed with distilled water, oven-dried (60 °C), and ground (30 mesh). The ground leaf material was stored in cold chamber at 4 °C for further analysis.

### 2.2. Conventional Analysis of Plant Leaves: Acid Digestion (AD) Method

For determination of P, K, Ca, Mg, S, Fe, Cu, Mn, and Zn, the ground samples were wet digested (using the nitro-perchloric acid method) [20,47] with modifications. The samples (0.5 g) were transferred to 50-mL glass digestion tubes and treated with 6 mL of acid solution (HNO_3_:HClO_4_ 2:1 v/v). Using a heating block digestion system, the samples were digested in three steps: i) heating at 120 °C for 30 min; ii) heating at 160 °C for 40 min; and iii) heating at 210 °C for 20 min. After digestion, the samples were cooled under laboratory conditions to room temperature (22 °C). After cooling, the samples were transferred to 50 mL volumetric flasks, then brought to volume with ultrapure water. Sub-samples were analyzed using a Ciros Vision ICP-OES (Spectro Analytical Instruments Inc., Kleve, Germany).

### 2.3. pXRF Analysis

**Figure 2:**
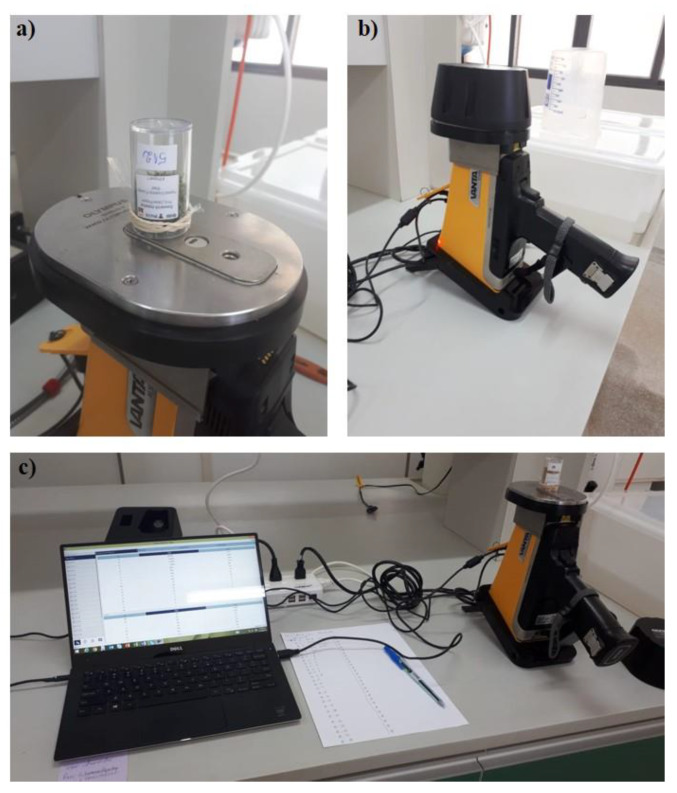
Details of portable X-ray fluorescence (pXRF) measurements. (**a**) Detail of ground leaf sample into the plastic vial and placed on X-ray source and detector aperture; (**b**) samples covered by a proper cap for protection against the X-ray; (**c**) data acquisition in real-time using a laptop connected to pXRF equipment.

Plant nutrients (P, K, Ca, Mg, S, Fe, Cu, Mn, and Zn) were also quantified via pXRF using an Olympus Vanta analyzer (Olympus^®^, Waltham, MA) equipped with a Rh tube (10–40 keV), silicon drift detector, and operated in the Geochem Mode on line power (115 VAC). Homogenized sub-samples of ground plant material were packed into 23-mL plastic vials (48.9 mm high and 27.6 mm diameter). Inside the plastic vials, at least 1 cm thickness of plant material was assured for analysis. The vials were covered using Prolene^®^ X-ray thin film (Chemplex Industries Inc., Palm City, FL, USA) and placed on the pXRF aperture. The X-ray thin film was held in place externally by latex rubber (Figure 2). The diameter of plastic vials was sufficient to cover the entire X-ray source and detector area without any influence of vial walls. The samples were scanned for 60 s as follows: beam 1 (first 30 s) for Fe, Cu, Mn, and Zn; beam 2 (last 30 s) for P, K, Ca, Mg, and S.

For quality assurance and quality control (QA/QC), the following materials were used: Olympus^®^ stainless calibration coin; blank sample (pure SiO_2_) and three certified reference materials (CRMs) from the National Institute of Standards and Technology (NIST 1547 peach leaves, NIST 1573a tomato leaves, and NIST 1515 apple leaves). Additionally, an internal standard prepared from soybean leaves was also employed. For each studied element, the recoveries (pXRF value/certified value) were obtained and linear regressions were made. The obtained equations were used as corrections factors (CF) of pXRF measurements. The limits of detection (LOD) considered for low-density sample types were (mg·kg^−1^): P (50); K (25); Ca (25); Mg (3000); S (50); Mn (5); Fe (5); Cu (5); and Zn (5).

### 2.4. Statistical Analyses

Descriptive statistics (maximum, minimum, median, average, and standard deviation) for the results obtained via both acid digestion (AD) and pXRF were calculated. For comparison between methods, correlations and simple linear regressions were performed for each crop. The crops with fewer than 10 samples (Table 1) were grouped and collectively termed “others” (e.g., vegetables, fruits, etc.).

For the full data set (n = 614), 70% of data were randomly selected to obtain linear regressions between AD and pXRF for each plant nutrient. The obtained equations were then validated using the remaining 30% of the data. The statistical significance of correlations and regressions was assessed based on Pearson’s correlation coefficient (R), determination coefficient of regression (R^2^), root mean square error (RMSE), and mean absolute error (MAE) (Equations (1) to (4), respectively). The linear regression analysis was performed using Sigma Plot Software version 14.0.

(1) $$R=\frac{\sum_{i=1}^{n}x_{i}y_{i}-\bar{x}\bar{y}}{\sqrt{\left[\sum_{i=1}^{n}x_{i}^{2}-n{\bar{x}}^{2}\right]\left[\sum_{i=1}^{n}y_{i}^{2}-n{\bar{y}}^{2}\right]}}$$

where n, $\bar{x}$, and $\bar{y}$ indicate the number of samples and the respective mean of each $x_{i}\;$ and $y_{i}$ variable.

(2) $$R^{2}=1-\frac{\sum_{i=1}^{n}{\left(Y_{i}-{\hat{Y}}_{i}\right)}^{2}}{\sum_{i=1}^{n}{\left(Y_{i}-{\bar{Y}}_{i}\right)}^{2}}$$

(3) $$RMSE=\sqrt{\overset{n}{\underset{i=1}{\sum }}\frac{{\left({\hat{Y}}_{i}-Y_{i}\right)}^{2}}{n}}$$

where, $Y_{i}$, ${\hat{Y}}_{i}$, and ${\bar{Y}}_{i}$ indicate the observed, the predicted, and the mean of the target variable.

(4) $$MAE=\frac{\sum_{i=1}^{n}\left|{\hat{y}}_{i}-y\right|}{n}$$

where n indicates the number of samples, and ${\hat{y}}_{i}$ and y indicate pXRF and AD measurements, respectively.

## 3. Results and Discussion

### 3.1. Recoveries of Elements

**Figure 3:**
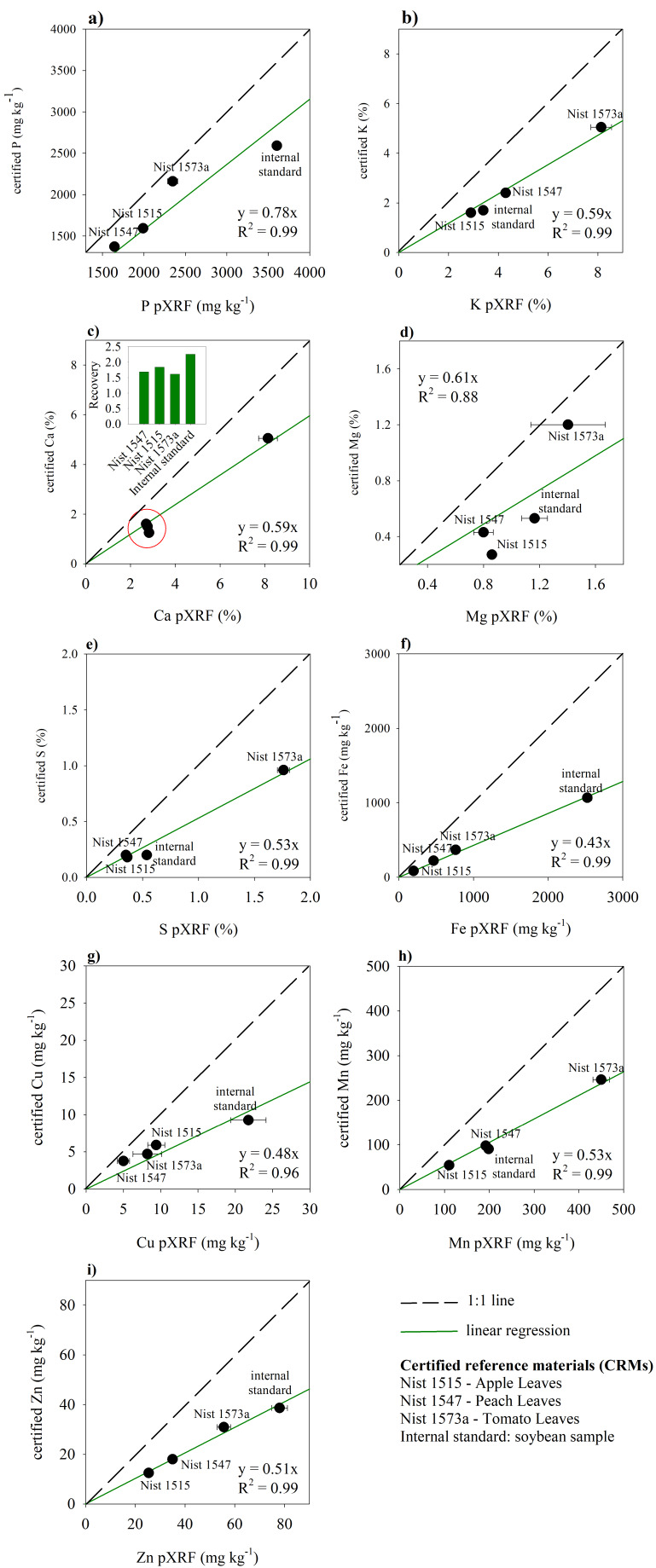
Calibration curve for obtained concentrations via pXRF and certified values for NIST 1515 (apple leaves), NIST 1547 (peach leaves), NIST 1537a (tomato leaves), and an internal standard (soybean sample): (**a**) Phosphorus; (**b**) Potassium; (**c**) Calcium; (**d**) Magnesium; (**e**) Sulfur; (**f**) Iron; (**g**) Copper; (**h**) Manganese; (**i**) Zinc.

For all elements, pXRF measurements were higher than certified values (Figure 3), supporting previous results [42]. A 1:1 straight correlation was not reached. However, the pXRF results were very reliable, since significant linear regressions between pXRF measurements and CRM values were obtained with very high R^2^ values (from 0.96 to 0.99) (Figure 3). The pXRF recoveries for each element were almost the same, independent of CRMs. The worst linear regression (R^2^ = 0.88) was obtained for Mg (Figure 3d), which produces low fluorescence energy and can be influenced by spectral interference. This limitation for Mg has been reported and its quantification via pXRF should be conducted under vacuum conditions [40].

For quality assurance and control, calibration routine on specific matrices can be performed for X-ray fluorescence analysis of plants [48]. Here, a calibration curve was obtained (Figure 3). The b parameter of linear equations (y = bx) (Figure 3) was used as the correction factor (CF) of pXRF measurements. The CF values represent the mean recoveries for four CRMs (NIST 1547; NIST 1573a; NIST 1515; and the internal standard for soybean leaves). For elemental assessment of thatch, deciduous leaves, grasses, tree bark, and herbaceous plants, CF values were considered as a mean of recoveries obtained for NIST 1515 and NIST 1547 [42]. The recovery assessment of CRMs is an important analytical procedure for quality assurance control of analysis [48], allowing a suitable interpretation and discussion of obtained results.

### 3.2. General Description of AD and pXRF Data

For all nutrients, the pXRF measurements were higher than LOD considered for low-density powder samples. The pXRF concentrations were higher than AD (Table 2). These results were expected, since the pXRF reports the total elemental concentration rather than the acid extractable concentration. The performance of AD methods is influenced by the digestion procedure and dilutions before quantification via ICP-OES [49,50,51]. Conversely, AD results higher than pXRF were found [35]. However, in Reference [35], they compared the AD method using ground and sieved leaf samples with the pXRF measurements performed directly on fresh leaves. The authors attributed the difference between pXRF and AD to the irregular distribution of nutrients in the leaves. The water content of fresh leaves can also be another factor influencing the results, since the water can attenuate X-rays [52] and underestimates the results [40].

Many factors can influence the plant analysis via X-ray fluorescence techniques [53]. The performance of pXRF is related to particle size distribution, uniformity, homogeneity, thickness, and water content [40,42,54,55]. In this work, the pXRF measurements were performed on ground (30 mesh) and sieved samples. A perfect uniformity of plant material size distribution was not expected. However, the size uniformity of plant materials had a minimal effect on chemical analysis via X-ray fluorescence techniques [54].

For all macro- and micronutrients, the mean values were higher than the mean adequate concentrations for plants (MACP) [55,56] (Table 2). Regarding the macronutrients, except for P, the percentage of samples with concentrations higher than the MACP ranged from 81% (Ca) to 90% (Mg). For P, only 38% of samples were higher than the MACP. For the micronutrients, the percentage of samples with concentrations exceeding the MACP ranged from 53% (Fe) to 67% (Zn). The most limiting nutrient was P, corroborating the very high P-adsorption capacity of highly-weathered leached soils (e.g., Oxisols) of Brazil [10,57,58], mainly by Fe oxides minerals. The mean concentrations of macronutrients decreased as K > Ca > Mg > S = P. For the micronutrients, concentrations were observed as Mn > Fe > Zn > Cu. In general, these results corroborate the expected uptake of nutrients by plants [55,56,59,60]. Plant mineral nutrition varies between species and from old to young leaves [55,56]. It is worthy to mention that in this work standard diagnostic leaves for nutritional status assessment were employed.

After N (not detectable by pXRF), Ca and K are the most abundant elements in plant dry matter, with concentrations ranging from 1 to 80 g kg^−1^ [56]. For Ca, both pXRF and AD methods (Table 2) resulted in similar mean values (13.64 and 14.15 g kg^−1^, respectively), and 81% of samples had Ca concentrations higher than the MACP [55]. The mean K concentrations varied slightly more between methods (24.20 and 18.12 g kg^−1^ for pXRF and AD, respectively), but both reported concentrations were considered appropriate. For K, 83% of samples exceeded the MACP (Table 2). Similar Ca and K concentrations in cowpea, maize, and mango leaves were also obtained via pXRF [40]. Sulfur is the macronutrient found in lower concentrations in plant dry matter, ranging from 1 to 15 g kg^−1^ [56,59]. The obtained results by both AD and pXRF (2.24 and 2.95 g kg^−1^, respectively) support this average concentration. These values were very similar to mean P concentrations (2.46 and 2.23 g kg^−1^, respectively, for AD and pXRF methods), which were below the adequacy level (3 to 5 g kg^−1^) [60].

High concentrations were observed for Mn, ranging from 21.73 to 4170 mg kg^−1^ when analyzed via pXRF and from 0.34 to 3273 mg kg^−1^ when analyzed via AD (Table 2). Adequate concentrations for foliar Mn in plants range from 30 to 500 mg kg^−1^, with a deficiency from 20 to 30 mg kg^−1^ and toxicity between 200 and 5300 mg kg^−1^, depending on the species [61]. The mean and median concentrations obtained for Fe via both AD and pXRF were within the range expected for plants (50 to 250 mg kg^−1^) but values up to 792 mg kg^−1^ were found via pXRF. Normally, Fe deficiency occurs at concentrations below 50 mg kg^−1^ [59]. Zinc concentration in plant dry matter is commonly at least five times higher than Cu [56]. Yet in this work, Cu concentrations in the leaves were much higher than those of Zn. It is possible that the low levels of Zn in the leaves are related to the antagonistic effect of Cu, which causes a reduction in Zn uptake [62].

### 3.3. Correlation between AD and pXRF

The obtained equations from linear regression (70% of full dataset) between AD and pXRF are shown in Table 3. Considering that AD methods are still a standard method for plant analysis, the AD data were plotted as a function of pXRF (AD = ***a*** + pXRF***x***). Except for Mg, high R values were obtained, ranging from 0.81 (K) to 0.98 (Cu). The R^2^ values ranged from 0.66 (K, S, and Fe) to 0.97 (Cu).

**Figure 4:**
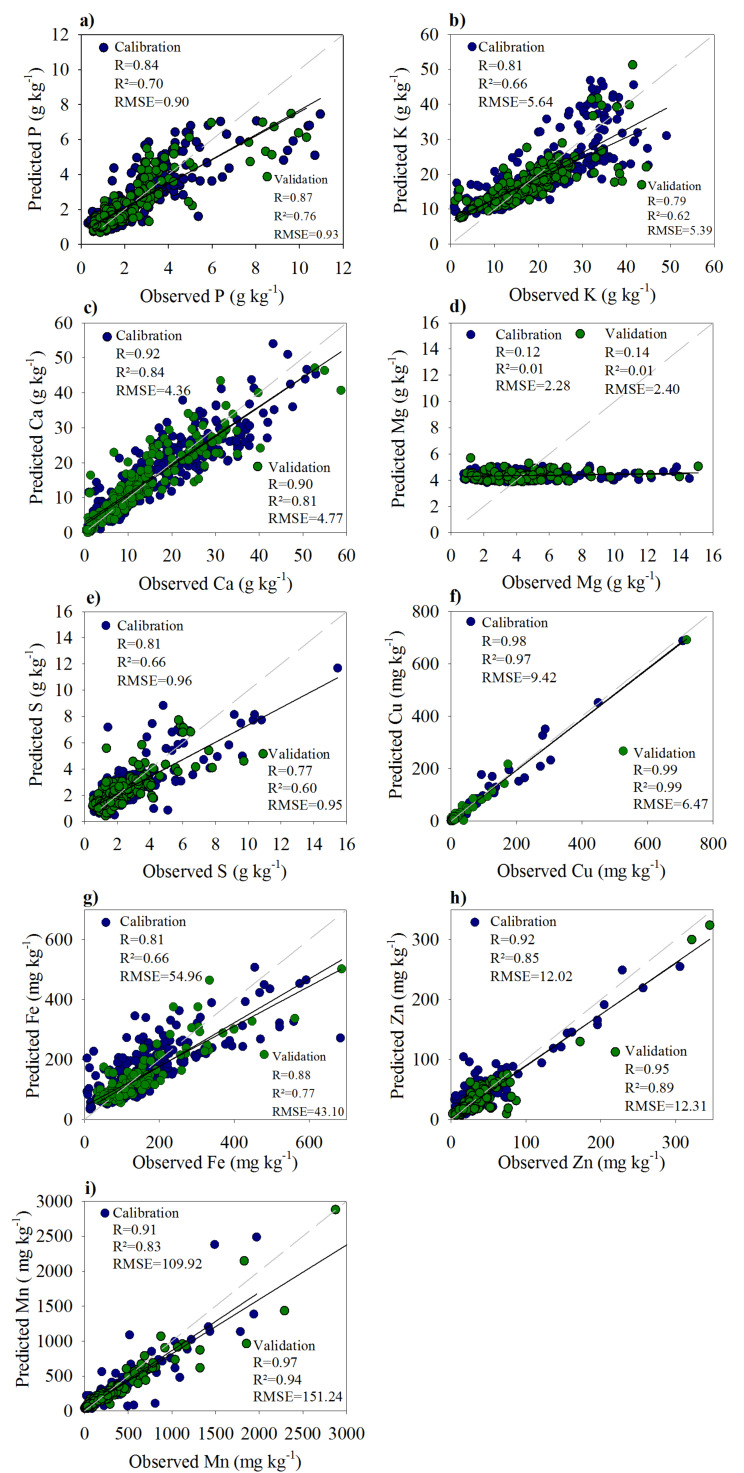
Prediction of macro- and micronutrients concentration in leaf samples from Brazilian crops (n = 614) using pXRF: (**a**) Phosphorus; (**b**) Potassium; (**c**) Calcium; (**d**) Magnesium; (**e**) Sulfur; (**f**) Copper; (**g**) Iron; (**h**) Zinc; (**i**) Manganese.

The non-significant correlation observed for Mg is related to the limitations for its determination via XRF techniques, as discussed before [40]. The validation of the obtained equations (Table 3) using 30% of the data revealed a very accurate prediction for all plant nutrients, with high R and R^2^ values (Figure 4). The best prediction was observed for Cu (R and R^2^ = 0.99; RMSE = 6.47 mg kg^−1^). Based on R and R^2^ values, the accuracy of predictions decreased in the following order: Cu > Mn > Zn > Ca > Fe > P > K > S.

Except for Ca, the micronutrients (heaviest elements) were better predicted than macronutrients (lightest elements). The ability of the X-ray fluorescence techniques to detect a particular element is directly related to its atomic number (*Z*) [63]. As the atomic number increases, so does the fluorescence energy. Thus, the so-called light elements (lighter than Ca; e.g., K, P, S, and Mg) are generally weakly identified and quantified via X-ray fluorescence techniques, while the heavier elements (Z > 20, e.g., Cu, Zn, Fe, and Mn) can be easily measured [22]. For the lightest elements (Mg, P, and S), the best performance of pXRF would be reached under vacuum conditions and without a Prolene^®^ film [40]. Even without specific and intricate vacuum conditions, suitable correlations between AD and pXRF were obtained (Figure 4). A strong correlation was obtained (R = 0.99) between pXRF and AD for S using pressed pellets of sugarcane leaves [46]. For P, a strong correlation between pXRF data and AD using CRMs for plants was also obtained [64].

The concentration of a given element in the sample can also influence the pXRF performance. For instance, Mn and Fe feature similar atomic numbers (Z = 25 and Z = 26, respectively), yet the prediction for Mn was better than Fe. As seen in Table 2, the Mn concentrations were higher than Fe. Similarly, Zn and Cu have similar atomic numbers (30 and 29, respectively). Better correlation was observed for Cu, as Cu concentrations were higher than Zn (Table 2). The adequate performance observed for Ca can also be related to its high concentration (Table 2). Similarly, a better correlation for Ca and K compared to Mn and Fe was found, where the total concentration within the plant was the compelling factor [65].

### 3.4. Correlation between pXRF and AD for Each Crop

**Figure 5:**
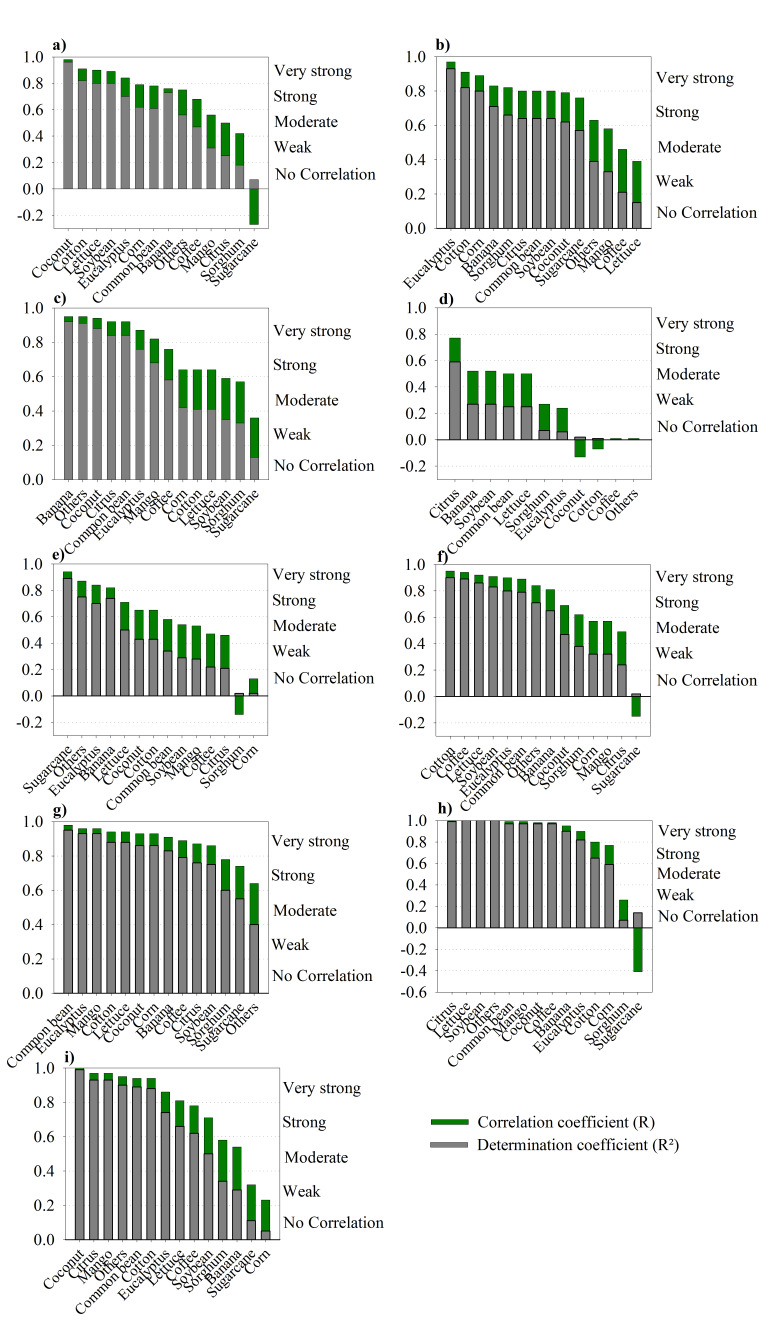
Correlation coefficient (R) and determination coefficient (R^2^) from the linear regression between pXRF and AD methods for each nutrient and crop. Citrus: orange and lemon. Others: pumpkin, garlic, cocoa, onion, cedar, grass, jackfruit, passion fruit, papaya, pepper, wheat, tomato, teak tree, and green bean. For Mg, there was no sufficient data for corn, mango, or sugarcane: (**a**) Phosphorus; (**b**) Potassium; (**c**) Calcium; (**d**) Magnesium; (**e**) Sulfur; (**f**) Iron; (**g**) Manganese; (**h**) Copper; (**i**) Zinc.

The correlation between pXRF and AD was nutrient- and crop-dependent (Figure 5). For P (Figure 5a), a very strong correlation between methods was observed for coconut, cotton, lettuce, soybean, and eucalyptus. Sugarcane showed no correlation for P. For K (Figure 5b), a very strong correlation was observed for eucalyptus, cotton, corn, banana, and sorghum and a weak correlation was shown for lettuce. Regarding Ca (Figure 5c), the correlation was strong and very strong for most crops and a weak correlation was also observed for sugarcane. As expected, the worst correlations were observed for Mg (Figure 5d), where only citrus had a strong correlation. For this nutrient, moderate correlations were observed for banana, soybean, common bean, and lettuce; weak correlations were observed for sorghum and eucalyptus; and no significant correlations were observed for coconut, cotton, and coffee. For S (Figure 5e), contrary to P and Ca, a very strong correlation was observed for sugarcane and no significant correlations were observed for sorghum and corn.

Regarding the micronutrients, very strong and strong correlations were observed for most crops, mainly for Fe, Mn, and Cu (Figure 5f–h). Corroborating the results for P and Ca, for all micronutrients, the worst correlation was observed in sugarcane. Regarding the macronutrients, the mean absolute errors (MAEs) were quite low (Table 4). The highest MAE values were observed for K (corn, lettuce, sorghum, and sugarcane), ranging from 14.57 to 35.39 mg kg^−1^ for these crops. For the micronutrients and considering all crops, the mean MAE decreased as Fe (74 mg kg^−1^) > Mn (56 mg kg^−1^) > Zn (7.4 mg kg^−1^) > Cu (5.1 mg kg^−1^).

Supporting the results found in this work, a strong correlation between pXRF and AD was also observed for Ca, Mn, Zn, and Cu in different plant species [44]. Assessing the elemental composition of plants (thatch, deciduous leaves, grasses, tree bark, and herbaceous plants) in mining-impacted areas, a significant correlation for Cu, Fe, Zn, Mn, Cd, Pb, and K was found [42]. Conversely, a poor correlation was observed for Cu in lettuce plants [41]. The results found that Ca, Cu, Zn, and Mn corroborate the performance of pXRF for soybean, wheat, corn, and cotton samples [37].

Different correlations between methods can be related to the intrinsic characteristics of plant materials, which will determine the performance of AD procedures [66]. The pXRF performance may be related to the anatomic characteristics of each plant species influencing the X-ray absorption and emission of the fluorescent energy. Further studies involving plant anatomy (e.g., epidermis, adaxial and abaxial surfaces, cuticle, stomata, and mesophyll) are still need to elucidate the diverse performance of pXRF for plant analysis. After that, in-field measurements will be greatly benefited. It is hypothesized here that plant materials with higher cellulose and lignin contents may be more difficult to digest. In general, grasses usually feature higher cellulose and lignin contents [67]. The acid digestion of a lettuce leaf or even the penetration of X-rays may be quite different when compared to more lignified leaves.

The accurate performance of pXRF to assess the elemental composition of plants will greatly contribute to fast and in-field diagnostic of nutritional status, improving the suitable management of soil fertility properties, food quality, and food security. Based on the results of this work, the foliar elemental composition can be analyzed via pXRF on ground and sieved leaf samples, eliminating the need for AD. For in-field applications, further studies are still needed to assess the other factors that can influence the pXRF results, such as water content, anatomy of leaves, cellulose, and lignin contents. Additionally, the nutritional status should be assessed combining pXRF with other proximal sensors (e.g., Vis-NIR, NixPro), as such techniques have been proven to enhance predictive models in coal and soils [68,69]. Especially for crops in which foliar fertilization is necessary, the pXRF can be a useful tool for decision-making. The assessment of fruit quality and nutritive value via pXRF is also promising [70] and worthy of additional study.

## 4. Conclusions

Except for Mg, pXRF spectrometry successfully quantified macro- and micronutrients in several leaf samples from important Brazilian crops. For many nutrients, a very strong correlation was observed between pXRF and the most traditional method for foliar analysis (nitro-perchloric digestion). The correlation between pXRF and acid digestion was nutrient- and crop-dependent. Except for Mg, a very strong correlation was always observed for eucalyptus leaves. Conversely, except for S and K, a weak or non-significant correlation was observed for sugarcane leaves.

Conventional foliar analysis based on acid digestion can be safely replaced by pXRF measurements made directly on ground and sieved leaf samples, dispensing the use of chemicals, acquisition, and maintenance of high-cost equipment (e.g., an atomic absorption spectrometer, an inductively coupled plasma-optical emission spectrometer). Measurements in the field directly on intact and fresh leaves still need more studies to elucidate all factors that can influence the pXRF performance for plant analysis.

## Figures and Tables

**Table 1:** Plant species (Brazilian crops) selected for this work.

Crop	Number of Samples (n)	
Cereals and Oilseeds (n = 157)		
Bean	*Phaseolus vulgaris*	45
Corn	*Zea mays*	14
Soybean	*Glycine max*	11
Sorghum	*Sorghum bicolor L.*	24
Wheat	*Triticum* spp	1
Cotton	*Gossypium hirsutum L.*	62
Fruits (n = 186)		
Banana	*Musa spp.*	96
Coconut	*Cocos nucifera L.*	53
Jackfruit	*Artocarpus heterophyllus*	1
Mango	*Mangifera indica*	26
Passion fruit	*Passiflora edulis*	2
Papaya	*Carica papaya*	8
Vegetables (n = 28)		
Garlic	*Allium sativum*	2
Green bean	*Phaseolus vulgaris L.*	1
Onion	*Allium cepa*	2
Tomato	*Solanum lycopersicum*	1
Lettuce	*Lactuca sativa*	14
Pumpkin	*Cucurbita* spp	7
Pepper	*Capsicum annuum*	1
Citrus (n = 46)		
Orange	*Citrus sinensis* *L. Osbeck*	7
Lemon	*Citrus limon*	39
Forest trees (n = 84)		
Cedar	*Cedrela fissilis*	5
Eucalyptus	*Eucalyptus globulus Labill*	78
Teak trees	*Tectona grandis L.f.*	1
Perennials and semi-perennials (n = 113)		
Coffee	*Coffea ssp.*	96
Cocoa	*Theobroma cacao*	1
Sugarcane	*Saccharum officinarum L.*	12
Grass	*Poaceae*	4

**Table 2:** Descriptive statistics (minimum, maximum, median, mean, and standard deviation) for pXRF and acid digestion (AD) data.

Nutrient	Minimum		Maximum		Median		Mean		s.d.		MACP	%
	pXRF	AD	pXRF	AD	pXRF	AD	pXRF	AD	pXRF	AD		
P (g kg^−1^)	0.52	0.32	8.99	10.96	1.67	1.64	2.46	2.23	1.75	1.71	2	38
K (g kg^−1^)	2.56	0.83	91.68	49.18	21.27	17.56	24.20	18.12	15.41	9.48	10	83
Ca (g kg^−1^)	2.08	0.48	55.49	58.74	13.96	10.80	13.64	14.15	9.94	10.96	5	81
Mg (g kg^−1^)**	4.34	0.79	19.34	15.09	8.36	3.89	8.62	4.30	2.35	2.34	2	90
S (g kg^−1^)	0.65	0.51	14.96	15.46	2.53	1.86	2.95	2.24	1.68	1.61	1	86
Fe (mg kg^−1^)	43.00	6.90	792.06	687.13	143.62	104.30	194.14	131.59	121.76	93.49	100	53
Cu (mg kg^−1^)	0.00	0.18	795.84	719.40	8.16	7.39	20.76	18.03	61.55	54.53	6	63
Mn (mg kg^−1^)	21.73	0.34	4170.04	3273.00	183.33	97.50	282.58	220.76	440.31	355.79	50	61
Zn (mg kg^−1^)	7.65	2.38	376.89	345.58	24.48	23.79	35.25	31.32	35.70	33.32	20	67

Mean adequate concentration for plant growth (MACP) [55]; ** The pXRF did not detect Mg in 36% of samples. For Mg, the descriptive statistics represents 64% of the full data set. %: percentage of samples with concentrations higher than MACP.

**Table 3:** Linear equations obtained for 70% of the full dataset correlating to pXRF and AD data.

Plant-Nutrient	Equation	R	R²
P	AD = 0.80pXRF + 0.27 *	0.84	0.70
K	AD = 0.49pXRF + 6.23 *	0.81	0.66
Ca	AD = 1.01pXRF − 2.24 *	0.92	0.84
Mg	AD = 0.13pXRF + 3.26 ^ns^	0.12	0.01
S	AD = 0.79pXRF − 0.08 *	0.81	0.66
Cu	AD = 0.87pXRF + 0.09 *	0.98	0.97
Fe	AD = 0.63pXRF + 7.76 *	0.82	0.66
Zn	AD = 0.59pXRF + 0.52 *	0.92	0.85
Mn	AD = 0.69pXRF + 18.73 *	0.91	0.83

* *p* < 0.01; non-significant (ns).

**Table 4:** The mean absolute error (MAE) between pXRF and AD methods for each nutrient and crop.

Crop	P	K	Ca	Mg	S	Cu	Fe	Zn	Mn
	----------------------(g kg^−1^) ----------------------					------------------- (mg kg^−1^)---------------------			
Banana	0.19	6.3	0.75	4.41	0.49	1.56	41.28	1.72	106.07
Citrus	0.24	0.24	2.94	1.55	0.01	0.37	66.22	1.58	30.39
Coconut	0.65	5.94	1.99	1.84	1.27	1.92	50.83	2.23	54.39
Coffee	0.01	4.73	4.5	3.49	0.67	5.91	44.13	3.2	48.86
Common bean	0.45	2.68	6.45	1.71	0.74	2.98	80.16	7.72	33.7
Corn	1.29	18.41	4.61	*	2.12	7.43	102.52	23.91	52.75
Cotton	0.37	0.51	1.43	5.73	1.02	1.45	26.89	1.64	33.08
Eucalyptus	0.58	3.37	3.65	5.64	0.52	1.77	96.6	5.55	153.14
Lettuce	2.16	32.59	3.63	2.49	1.82	17.37	91.2	14.24	56.18
Mango	0.33	0.76	5.19	*	0.24	4.28	31.75	1.51	40.31
Sorghum	2.17	35.39	4.24	8.17	1.95	5.69	194.55	22.42	32.72
Soybean	0.81	3.5	6.05	0.9	0.88	15.78	99.18	10.27	55.21
Sugarcane	0.41	14.57	2.96	*	0.18	2.16	67.59	6.13	50.29
Others	0.42	6.85	0.7	6.39	0.45	3.34	47.64	1.14	30.79
Mean	0.72	9.70	3.51	3.85	0.88	5.14	74.32	7.38	55.56

Citrus: orange and lemon. Others: pumpkin, garlic, cocoa, onion, cedar, grass, jackfruit, passion fruit, papaya, pepper, wheat, tomato, teak tree, and green bean. *For Mg, there was no sufficient data for corn, mango, or sugarcane.

## Supplementary Materials

The following are available online at https://www.mdpi.com/1424-8220/20/9/2509/s1, Table S1: Sampling details of diagnostic leaves of each crop selected for this study.
