# Supplementary material for: Foliar Elemental Analysis of Brazilian Crops via Portable X-ray Fluorescence Spectrometry
Source: Sensors (Basel). 2020 Apr 29;20(9):2509. doi: 10.3390/s20092509 (PMC7249210; doi:10.3390/s20092509)
Supplement: Supplementary file 1 [file sensors-20-02509-s001.pdf]

sensors

Supplementary Material

Foliar Elemental Analysis of Brazilian Crops via Portable X-ray Fluorescence Spectrometry

Camila S. Borges [1], David C. Weindorf [2], Geila S. Carvalho [1], Luiz R. G. Guilherme [1], Thalita Takayama [1], Nilton Curi [1], Geraldo J. E. O. Lima [3] and Bruno T. Ribeiro [1,2,*]

[1] Department of Soil Science, Federal University of Lavras – UFLA, Doutor Sylvio Menicucci Avenue, Lavras, Minas Gerais State, 37200-900, Brazil; camila.borges@estudante.ufla.br (C.S.B); geilacarvalho@ufla.br (G.S.C.); guilherm@ufla.br (L.R.G.G.); thalita.takayama@estudante.ufla.br (T.T.); niltcuri@ufla.br (N.C.)

[2] Department of Plant and Soil Science, Texas Tech University, Bayer Plant Science Building, Room 211A, 2911 15th Street, Lubbock, TX 79409-2122; david.weindorf@ttu.edu

[3] Campo – Environmental and Technological Agricultural Center, Lindolfo Garcia Adjuto Street, 1000, Paracatu, Minas Gerais State, 38600-000, Brazill; geraldo.lima@campoanalises.com.br

* Correspondence: bruno.ribeiro@ttu.edu; brunoribeiro@ufla.br

Received: 25 March 2020; Accepted: 23 April 2020; Published: 29 April 2020

Table S1. Sampling details of diagnostic leaves of each crop selected for this study [11].

Crop	Criteria
Banana	10 central centimeters of the third leaf from the apex without the central rib and peripheral halves
Cedar	Randomly
Citrus	Third of fourth leaf of branches with fruits
Cocoa	Third leaf from the apex of launch
Coconut	Three leaflets on each side of the central part of the fourteen leaf (adult plants) or ninth leaf (young plants)
Coffee	Third and fourth pairs of leaves from the apex of productive branches at medium height
Common bean	Leaves of the middle third
Corn	Opposite leaf and below the cob without the central third
Cotton	Fifth leaf from the apex
Eucalyptus	Freshly ripe leaves of primary branches
Garlic	Newer leaf fully developed
Grass	Freshly ripe leaves or from all positions in the shoot
Green bean	Fourth leaf from the tip
Jackfruit	Randomly
Lettuce	Freshly ripe leaves
Mango	Leaves in different positions in the canopy
Onion	Highest leaf
Papaya	Leaf with the first flower fully expanded
Passion Fruit	Fourth leaf from the tip of middle branches
Pepper	Sixth leaf petiole from the tip
Pumpkin	Petioles of new leaves completely expanded.
Sorghum	Leaves in middle position on the plant
Soybean	Third leaf from the apex on the main stem, with petiole
Sugarcane	Leave +3 in the insertion region of thatch sheath. 20 cm leaf center
Teak trees	Randomly
Tomato	Petiole/Limbo of the opposite leaf the third bunch
Wheat	First to fourth leaf from top of plant
